# Forensic and genetic characterizations of diverse southern Thai populations based on 15 autosomal STRs

**DOI:** 10.1038/s41598-021-04646-1

**Published:** 2022-01-13

**Authors:** Metawee Srikummool, Suparat Srithawong, Kanha Muisuk, Sukrit Sangkhano, Chatmongkon Suwannapoom, Jatupol Kampuansai, Wibhu Kutanan

**Affiliations:** 1 Department of Biochemistry, Faculty of Medical Science, Naresuan University, Phitsanulok, 65000 Thailand; 2 Department of Biology, Faculty of Science, Khon Kaen University, Khon Kaen, 40002 Thailand; 3 Department of Forensic Medicine, Faculty of Medicine, Khon Kaen University, Khon Kaen, 40002 Thailand; 4 School of Public Health, Walailak University, Nakhon Si Thammarat, 80161 Thailand; 5 School of Agriculture and Natural Resources, University of Phayao, Phayao, 56000 Thailand; 6 Department of Biology, Faculty of Science, Chiang Mai University, Chiang Mai, 50202 Thailand; 7 Research Center in Bioresources for Agriculture, Industry and Medicine, Chiang Mai University, Chiang Mai, 50202 Thailand

**Keywords:** Genetic variation, Structural variation, Genotyping and haplotyping, PCR-based techniques

## Abstract

Southern Thailand is home to various populations; the Moklen, Moken and Urak Lawoi’ sea nomads and Maniq negrito are the minority, while the southern Thai groups (Buddhist and Muslim) are the majority. Although previous studies have generated forensic STR dataset for major groups, such data of the southern Thai minority have not been included; here we generated a regional forensic database of southern Thailand. We newly genotyped common 15 autosomal STRs in 184 unrelated southern Thais, including all minorities and majorities. When combined with previously published data of major southern Thais, this provides a total of 334 southern Thai samples. The forensic parameter results show appropriate values for personal identification and paternity testing; the probability of excluding paternity is 0.99999622, and the combined discrimination power is 0.999999999999999. Probably driven by genetic drift and/or isolation with small census size, we found genetic distinction of the Maniq and sea nomads from the major groups, which were closer to the Malay and central Thais than the other Thai groups. The allelic frequency results can strength the regional forensic database in southern Thailand and also provide useful information for anthropological perspective.

## Introduction

Southern Thailand lies on the Malay Peninsula, bordering the Gulf of Thailand to the East, the Andaman Sea to the West, and Malaysia to the South. A census size of ~ 9.16 million in southern Thailand is about 13.35% of the total census size of the country (68.61 million in 2020)^1^. Most people are southern Thai Buddhists (66%) and southern Thai Muslims (33%), while the minorities, e.g. sea nomad and Maniq groups account for about 0.33%^2^. The populations of the three groups of sea nomads are 4000, 2000 and 3000 for Moklen, Moken and Urak Lawoi’, respectively, while only 250 were recorded for the Maniq^1,2^. The languages spoken in southern Thailand belonging to three linguistic families: Tai-Kadai (TK), Austroasiatic (AA) and Austronesian (AN). The AA-speaking Maniq who are scattered through the jungle are regarded as the indigenous people of Southeast Asia or often referred to “negritos” because of their phenotypic difference and traditional mode of subsistence practice that is hunter-gatherers^3^. The AN-speaking sea nomads were used to subsist through maritime foraging in most of the year, although nowadays they prefer to settle in the coastal area of Thailand and Myanmar^4^. Both Maniq and sea nomads were minority groups and thought to be native in southern Thailand since prehistorical time, together with the other groups, e.g. AA-speaking Mon and Khmer before the occupation of the AN-speaking Malays and TK-speaking Thais, thought the Mon and Khmer people are nowadays disappear in southern Thailand^5^.

The autosomal short tandem repeats (STRs) show a number of advantages for both population genetic and forensic studies, i.e. distribution across the human genome which led to escape from natural selection, highly polymorphism and informativeness to distinguish recently diverged populations^6,7^. In Thailand, studies on forensic microsatellites and also other markers have focused on northern, northeastern and central Thailand leaving the southern region understudied^8–12^. The only one study on autosomal STRs in southern Thailand indicated that the Thai-Malay Muslim and Thai Buddhist who lived in the five deep Southern Thai provinces had non-significant genetic difference^13^.

In addition, there were some other genetic studies on southern Thai populations using uni-parentally inherited markers^3,4^. The mitochondrial (mt) DNA investigation of the Moken showed ancient basal mtDNA haplogroup M21d and M46 with very low genetic diversity^4^. The basal mtDNA haplogroup M21a, R21 and M17a and Y chromosomal haplogroup K were also observed in the Maniq as well as close genetic affinity between the Maniq and other indigenous people of Southeast Asia in Malaysia, reflecting an ancient ancestry of the Maniq and common genetic ancestry of indigenous people of Southeast Asia in the Malay Peninsula^3^.

To expand the genetic studies in southern Thailand, we reported genotypes of 15 autosomal STRs of seven southern Thai populations: one AA-speaking Maniq, four AN-speaking Moklen, Moken, Urak Lawoi’ and southern Thai Muslim and two TK-speaking southern Thai Buddhist and southern Thai Takbai. We explored genetic structure and relationships of southern Thai populations with other Thai and Malaysian populations^8,10–14^. In addition, because the forensic database combined diverse southern Thai populations has not yet been established, we created a regional DNA database of 15 autosomal STRs of southern Thailand.

## Results and discussions

### Genetic diversities and forensic parameters

Raw genotypic data of 15 STRs of 334 southern Thai samples are provided in Table S1. Total genetic diversity of all southern Thai samples was 0.7871 ± 0.3945, whereas that in individual populations ranged from 0.6742 ± 0.3526 in the Maniq to 0.7943 ± 0.4012 in southern Thai Buddhist (Table 1). The reduced genetic diversity of the Maniq is possibly driven by genetic drift associated with geographic isolation and very small population sizes, as reported previously^3^. When the genetic diversity calculated form the same marker set was compared between two hunter-gatherer groups in Thailand, the Maniq from the South had diversity value greater than the Mlabri from the North (0.547 ± 0.288)^15^ although the sample size of Maniq (*n* = 15) is lower than the Mlabri (*n* = 19). Also the genetic diversity results of these 15 STRs of ~ 70 Thai populations^8–13,15^ revealed that the Mlabri had the lowest genetic diversity, indicating a strong genetic drift of the Mlabri. Regarding the sea nomads and excluding the Moken due to their small sample size, the Moklen and Urak Lawoi’ showed lower genetic diversity than other Thai and Malaysian populations (Table 1), reflecting certain degree of genetic drift.

**Table 1:** General information and results on genetic diversities of the studied and compared populations.

Population	Code	Sample size	Location	Language	References	Average H_E_	Total allele	Gene diversity (S.D.)
Southern Thai Takbai	JH	40	Southern Thailand	Tai-Kadai	Present study	0.7826	117	0.7786 (0.3949)
Southern Thai Muslim	MST	22	Southern Thailand	Austronesian	Present study	0.7903	109	0.7759 (0.3994)
Southern Thai Buddhist	BST	52	Southern Thailand	Tai-Kadai	Present study	0.7963	125	0.7943 (0.4012)
Maniq	MN	15	Southern Thailand	Austroasiatic	Present study	0.6742	73	0.6742 (0.3526)
Moklen	MLK	22	Southern Thailand	Austronesian	Present study	0.7535	97	0.7535 (0.3869)
Urak Lawoi’	UL	29	Southern Thailand	Austronesian	Present study	0.7532	102	0.7469 (0.3816)
Moken	MOK	4	Southern Thailand	Austronesian	Present study	0.7238	55	0.7238 (0.4202)
Southern Thai Muslim	MUS	104	Southern Thailand	Austronesian	^13^	0.7876	137	0.7860 (0.3953)
Southern Thai Buddhist	BUD	46	Southern Thailand	Tai-Kadai	^13^	0.7858	121	0.7837 (0.3966)
Malay	ML1	110	Malaysia	Austronesian	^14^	0.7942	144	0.7942 (0.3991)
Malay	ML2	246	Malaysia	Austrone sian	^14^	0.7962	163	0.7962 (0.3990)
Yuan	YU	135	Northern Thailand	Tai-Kadai	^8^	0.7839	136	0.7839 (0.3939)
Yong	YO	55	Northern Thailand	Tai-Kadai	^8^	0.7758	125	0.7758 (0.3922)
Central Thai	CT	246	Central Thailand	Tai-Kadai	^12^	0.7916	151	0.7854 (0.3939)
Mon	MO	92	Central Thailand	Austroasiatic	^12^	0.7913	137	0.7745 (0.3901)
Khmer	KH	48	Northeastern Thailand	Austroasiatic	^11,37^	0.7589	114	0.7589 (0.3846)
Lao Isan	IS	272	Northeastern Thailand	Tai-Kadai	^11^	0.7844	167	0.7669 (0.3851)

When genotype data of total 334 southern Thai samples were combined and calculated the allelic frequency for the 15 STR loci (Table 2), there are two loci (*D19S433* and *D18S51*) that depart from the Hardy–Weinberg equilibrium (HWE) even after applying Bonferoni adjustment (*p* < 0.0033). Although the forensic parameters show that both loci are highly discriminating (power of discrimination (PD) = 0.9246 for *D19S433* and 0.9513 for *D18S51*) and power of exclusion (PE) = 0.5757 or *D19S433* and 0.6873 for *D18S51*)), the lack of HWE must be taken into account in forensic investigation. A total of 157 alleles were detected, ranging from 6 alleles at *TPOX* to 21 alleles at *FGA*. The maximum allele frequencies is observed in *TPOX* (0.5472). The lowest expected heterozygosity (*H*_*E*_) was observed in the *TPOX* (0.6201), while the highest *H*_*E*_ was in the *FGA* (0.8690) (Table 2). The polymorphic information content (PIC) ranged from 0.5672 (*TPOX*) to 0.8529 (*D2S1338*) and matching probability (MP) values are from 0.0374 (*FGA*) to 0.2037 (*TPOX*) (Table 2). The power of discrimination (PD) ranged from 0.7963 (*TPOX*) to 0.9673 (*D2S1338*) (Table 2), with a value of 0.9999999999999999 for the combined PD. The power of exclusion (PE) ranged from 0.3121 (*D3S1358*) to 0.7588 (*FGA*) (Table 2), with a combined PE value of 0.99999622.

**Table 2:** Allele frequencies of total southern Thais based on the 15 autosomal STR loci (*n* = 334).

Allele	*D8S1179*	*D21S11*	*D7S820*	*CSF1PO*	*D3S1358*	*THO1*	*D13S317*	*D16S539*	*D2S1338*	*D19S433*	*VWA*	*TPOX*	*D18S51*	*D5S818*	*FGA*
6						0.1199									
7			0.0195			0.2849						0.0015		0.0180	
8	0.0060		0.2628	0.0015		0.1229	0.3584	0.0135				0.5472		0.0390	
9			0.0766	0.0150		0.3613	0.1009	0.1512		0.0075		0.1439			
9.3						0.0570									
10	0.1362		0.1757	0.2096		0.0495	0.1431	0.1347				0.0405	0.0045	0.2444	
11	0.0734		0.2808	0.2859		0.0045	0.2063	0.3220		0.0060	0.0030	0.2429	0.0045	0.2684	
12	0.1332		0.1547	0.3982	0.0030		0.1566	0.2246		0.0510		0.0240	0.1078	0.2729	
12.2										0.0075					
13	0.1826		0.0255	0.0704	0.0015		0.0301	0.1287		0.2144	0.0045		0.1228	0.1409	
13.2										0.0390					
14	0.1991		0.0015	0.0105	0.0526		0.0030	0.0210		0.2594	0.2054		0.1542	0.0165	
14.2										0.1005					
15	0.1677		0.0030	0.0090	0.3083		0.0015	0.0045		0.0960	0.0420		0.2500		0.0015
15.2										0.1694					
16	0.0793				0.3353				0.0075	0.0270	0.1319		0.1632		0.0015
16.2										0.0195					
17	0.0210				0.2211				0.1124	0.0015	0.3253		0.0644		
17.2										0.0015					
18	0.0015				0.0767				0.0930		0.1829		0.0434		0.0060
19					0.0015				0.2069		0.0855		0.0269		0.0663
20									0.1139		0.0180		0.0284		0.0738
20.2															0.0045
21									0.0225		0.0015		0.0105		0.1852
21.2															0.0120
22									0.0570				0.0150		0.1807
22.2															0.0045
23									0.1814				0.0030		0.1446
23.2															0.0075
24									0.1214						0.1581
24.2															0.0105
25									0.0660				0.0015		0.0904
25.2															0.0015
26									0.0180						0.0316
26.2															0.0015
27		0.0060													0.0136
28		0.0465													0.0030
28.2		0.0015													
29		0.2549													
30		0.2369													
30.2		0.0300													
30.3		0.0015													
31		0.0705													0.0015
31.2		0.0840													
32		0.0165													
32.2		0.1574													
32.3		0.0015													
33		0.0015													
33.2		0.0765													
34.2		0.0150													
No. alleles	10	15	9	8	8	7	8	8	11	14	10	6	15	7	21
*H*_*O*_	0.8204	0.8198	0.7598	0.6856	0.7078	0.7147	0.7613	0.7934	0.8499	0.8168	0.7898	0.6547	0.8264	0.7598	0.8852
*H*_*E*_	0.8517	0.8342	0.7916	0.7115	0.7363	0.7543	0.7737	0.7889	0.8683	0.8348	0.7932	0.6201	0.8537	0.7730	0.8690
HWE	0.1551	0.5497	0.4782	0.0452	0.7972	0.3155	0.1199	0.2903	0.7775	0.0004	0.0414	0.0060	0.0027	0.9449	0.1458
MP	0.0592	0.0543	0.0738	0.1392	0.1787	0.0953	0.0874	0.0821	0.0327	0.0754	0.0879	0.2037	0.0487	0.0888	0.0374
PD	0.9408	0.9457	0.9262	0.8608	0.8213	0.9047	0.9126	0.9179	0.9673	0.9246	0.9121	0.7963	0.9513	0.9112	0.9626
PIC	0.8020	0.8069	0.7578	0.6533	0.5884	0.7164	0.7393	0.7538	0.8529	0.7760	0.7415	0.5672	0.8317	0.7348	0.8492
PE	0.6462	0.6224	0.5241	0.3978	0.3121	0.4514	0.5281	0.5834	0.6946	0.5757	0.5455	0.3616	0.6873	0.5306	0.7588
TPI	2.8571	2.6667	2.0688	1.5619	1.3065	1.7526	2.0886	2.3986	3.3300	2.3509	2.1786	1.4478	3.2500	2.1013	4.2368

No. alleles, number of allele; H_O_, observed heterozygosity; H_E_, expected heterozygosity; HWE, Hardy–Weinberg *p* value; MP, matching probability; PD, power of discrimination; PIC, po ly mo r p h ic i nformation content; power of discrimination; TPI, total paternity index; GD, gene diversity; CMP, combined matching probability; CPD, combine power d isc rim in ation; CPE, combined pow er discrimination.

### Genetic relatedness and genetic structure of southern Thai populations

**Figure 1:**
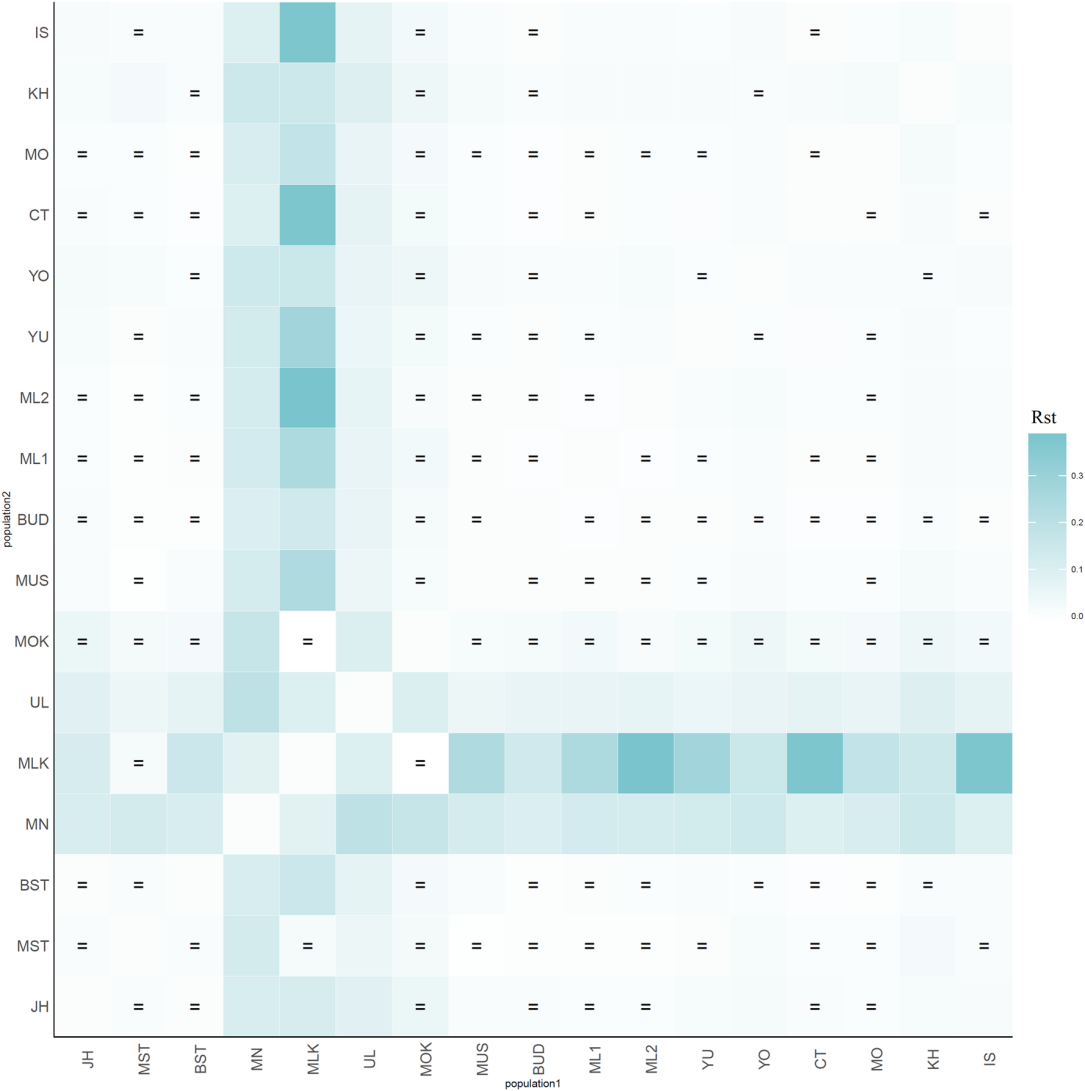
Heat plot of *R*_*st*_ values between total 17 populations. The “ = ” symbol indicates non-significance of *R*_st_ values (*p* > 0.05).

**Figure 2:**
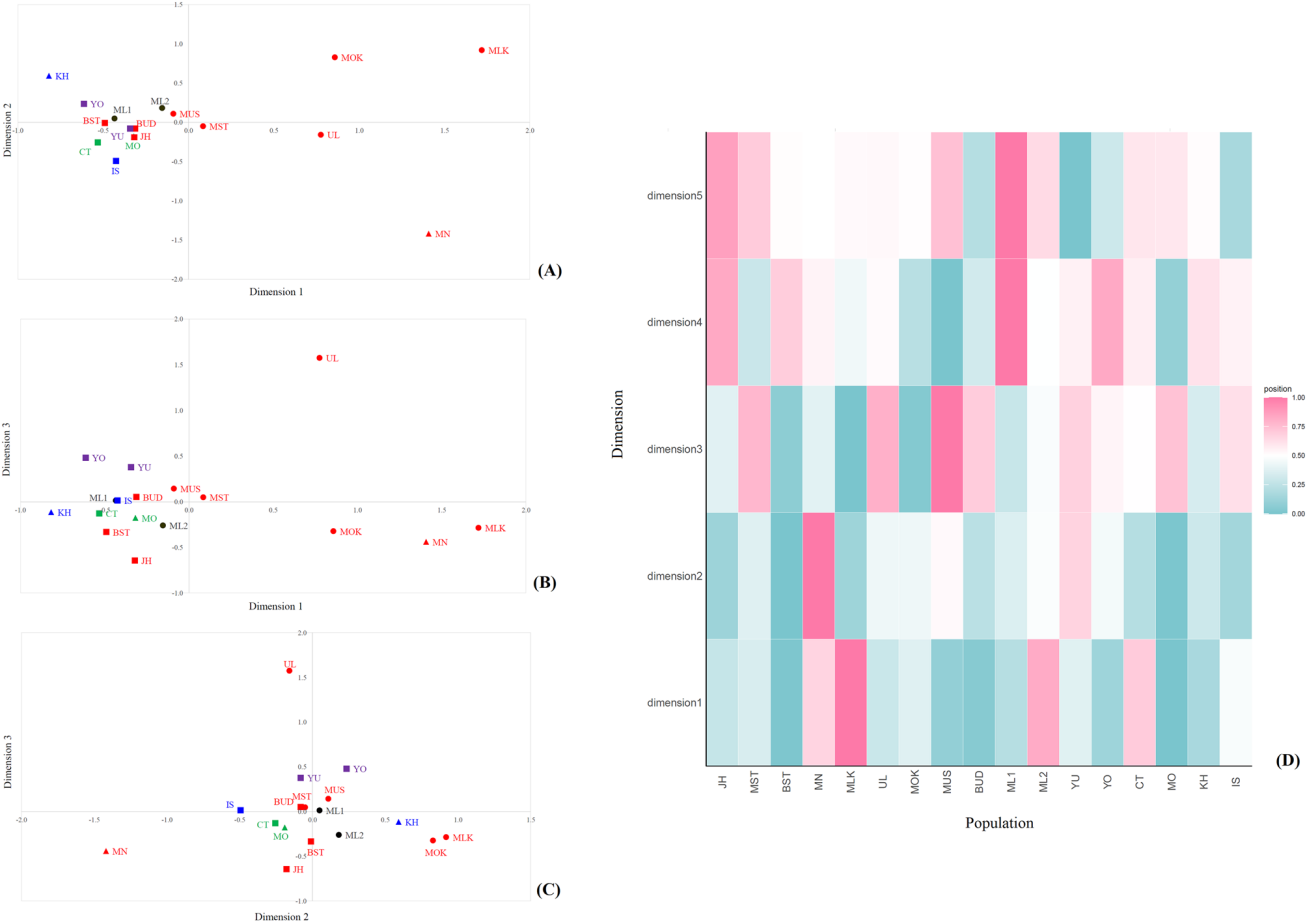
The three-dimensional MDS plots for 17 populations (**A**–**C**) (stress = 0.0030) and the heat plot of standardized values of MDS with five dimensions (D). See population abbreviation in Table 1. Red, purple, green, blue and black indicate populations from southern Thailand, northern Thailand, northeastern Thailand, central Thailand and Malaysia, respectively. Circle, square and triangle indicate Austronesian, Tai-Kadai, Austroasiatic families, respectively.

One measure of genetic relationship among populations was a genetic distance value. The result of genetic distance (*R*_st_) among 17 Thai and Malaysian populations showed that the Maniq (MN) and Urak Lawoi’ (UL) were genetically different from each other and from other populations (Fig. 1) whereas the Moklen (MLK) showed significantly difference from almost all comparisons (*p* > 0.05), except with the pairs of newly generated southern Thai Muslim (MST) and Moken. However, due to the effect of very small sample size, the Moken did not differ from almost populations. In general, the Maniq and sea nomads from southern Thailand exhibited genetic differentiation from the other groups. Then, the matrix of *R*_st_ were constructed to multi-dimensional scaling (MDS) plots. The three-dimensional MDS result based on dimension 1 and 2 showed genetic distinction of Maniq (MN) and three sea nomads, i.e. Moklen (MLK), Moken (MOK) and Urak Lawoi’ (UL) from the other groups from Thailand and Malaysia. The MDS analysis based on dimensions 3 showed genetic differences of Urak Lawoi’ from other populations (Fig. 2A–C). The heat plot of the MDS indicated genetic distinction of Moklen and Maniq in dimension 1 and 2, respectively and genetic difference of Urak Lawoi’ from other sea nomads in dimension 3 (Fig. 2D).

**Figure 3:**
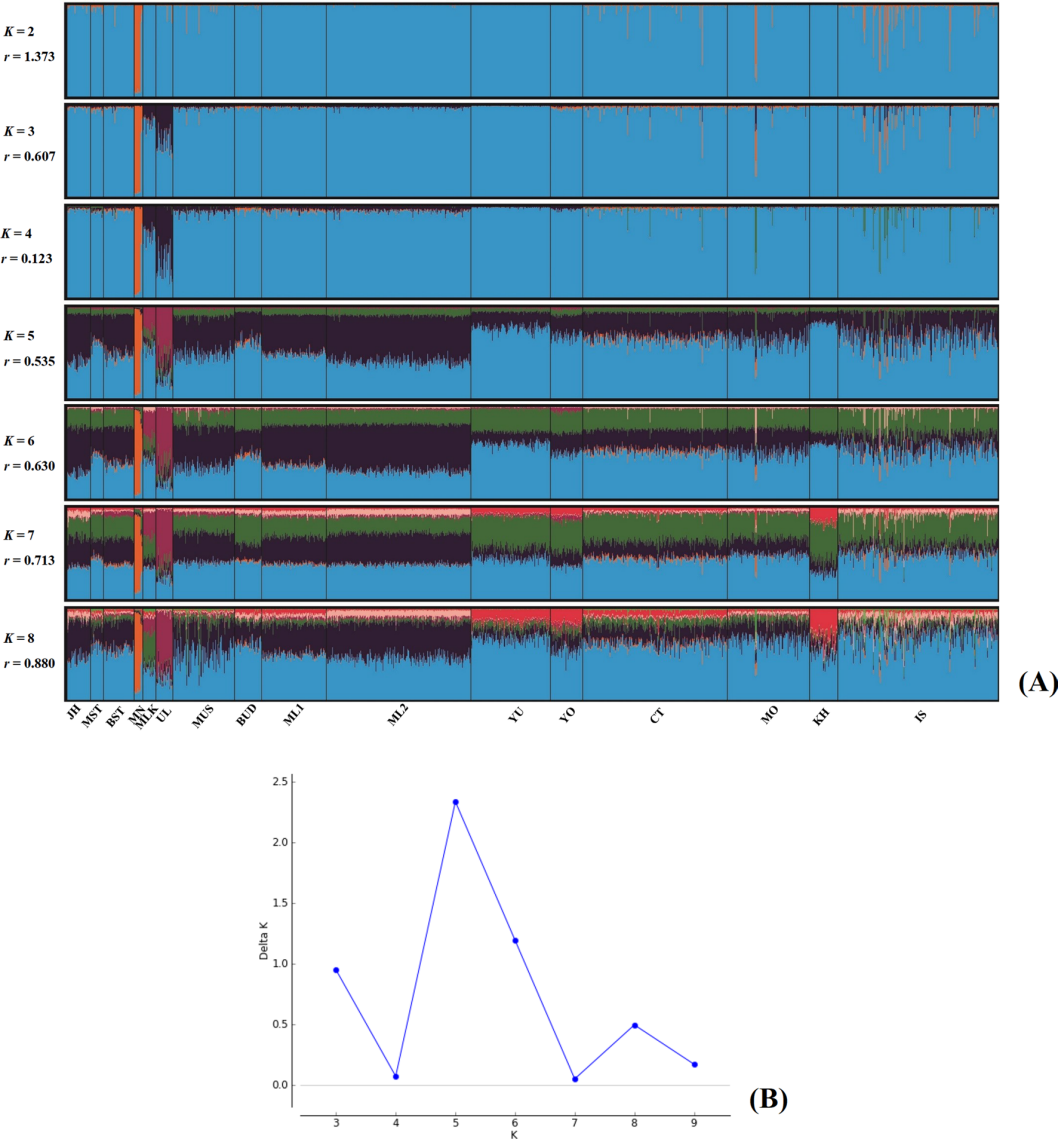
STRUCTURE result at *K* = 2–8 (**A**). Each individual is represented by a single column that is divided into segments whose size and color correspond to the relative proportion of a particular cluster. Populations are separated by black lines and population codes are listed in Table 1. Number of populations with the highest posterior probability expressed as the Delta *K* (**B**).

To further explore cryptic population structure and genetic relationship among 16 populations without the Moken by STRUCTURE, we present the result of *K* from 2 to 8 (Fig. 3A) and at *K* = 5 which is the suitable cluster (Fig. 3B)^16^. The first cluster was in the Maniq (MN), as represented by orange, while the second clusters (purple) stood out in the sea nomads: Moklen (MLK) and Urak Lawoi’ (UL), supporting their genetic uniqueness (Fig. 3A). The other three clusters (dark blue, light blue and green) were distributed in all populations at different proportions: (1) the dark blue component greatly emerged in southern Thais (MST, MUS, BST and BUD), Malays (ML1 and ML2), populations from central Thailand (MO and CT), (2) light blue strongly emerged in the other Thais from northern (YO and YU) and northeastern regions (IS and KH) and the green component was roughly distributed in all populations, except for a reduction in the Maniq and Urak Lawoi’. Interestingly, although the Moklen and Urak Lawoi’ occupy their own cluster (purple), the Moklen exhibited mixed ancestries compared to the Urak Lawoi’ (Fig. 3A), indicating stronger interactions between Moklen and the other populations.

Overall, there were three main observations according to genetic relationship results. First, the Maniq and sea nomads exhibited extremely genetic differences from other Thai and Malaysian populations. The distinct genetic structure coupled with low genetic diversity (Table 1) is probably driven by genetic drift and/or inbreeding due to their geographical isolation and small census size. Reduced genetic diversity of the Maniq was also observed in previous study of mtDNA and Y chromosomal variations^3^. Second, among the sea nomad groups (excluding the Moken), the Urak Lawoi’ and Moklen showed genetic dissimilarity with the latter displaying genetic admixture with other populations. According to ethnolinguistic background, the Moklen are more closely related to the Moken and both of them are more distant from Urak Lawoi’^4^. Although languages of sea nomads were grouped within Austronesian family and Malayo-Polynesian sub-family, different in dialects were spoken; the Urak Lawoi’ or Orang Laut speak Malayic that distantly related to both Moken and Moklen who shared many cultural connections. In addition, the Urak Lawoi’ was culturally isolated but the Moklen had frequently interacted with and influenced by other southern Thais^1,17,18^. Therefore, the unique genetic signature of Urak Lawoi’ and mixed ancestries of Moklen could be described by ethnolinguistic and cultural evidence. Third, we found more genetic similarity between major southern Thais and populations from central Thailand than other regions. The present result was in agreement with a recent genome-wide study^19^ that could be explained by historical evidence; there were movements from the central region to the south during the Ayutthaya Period (during 1350–1767 A.D.)^20^ and genetic admixture between the southern Thai and Malays after the settlement period might be possible^13^.

### Genetic relationships between southern Thai populations and other Asian populations

**Figure 4:**
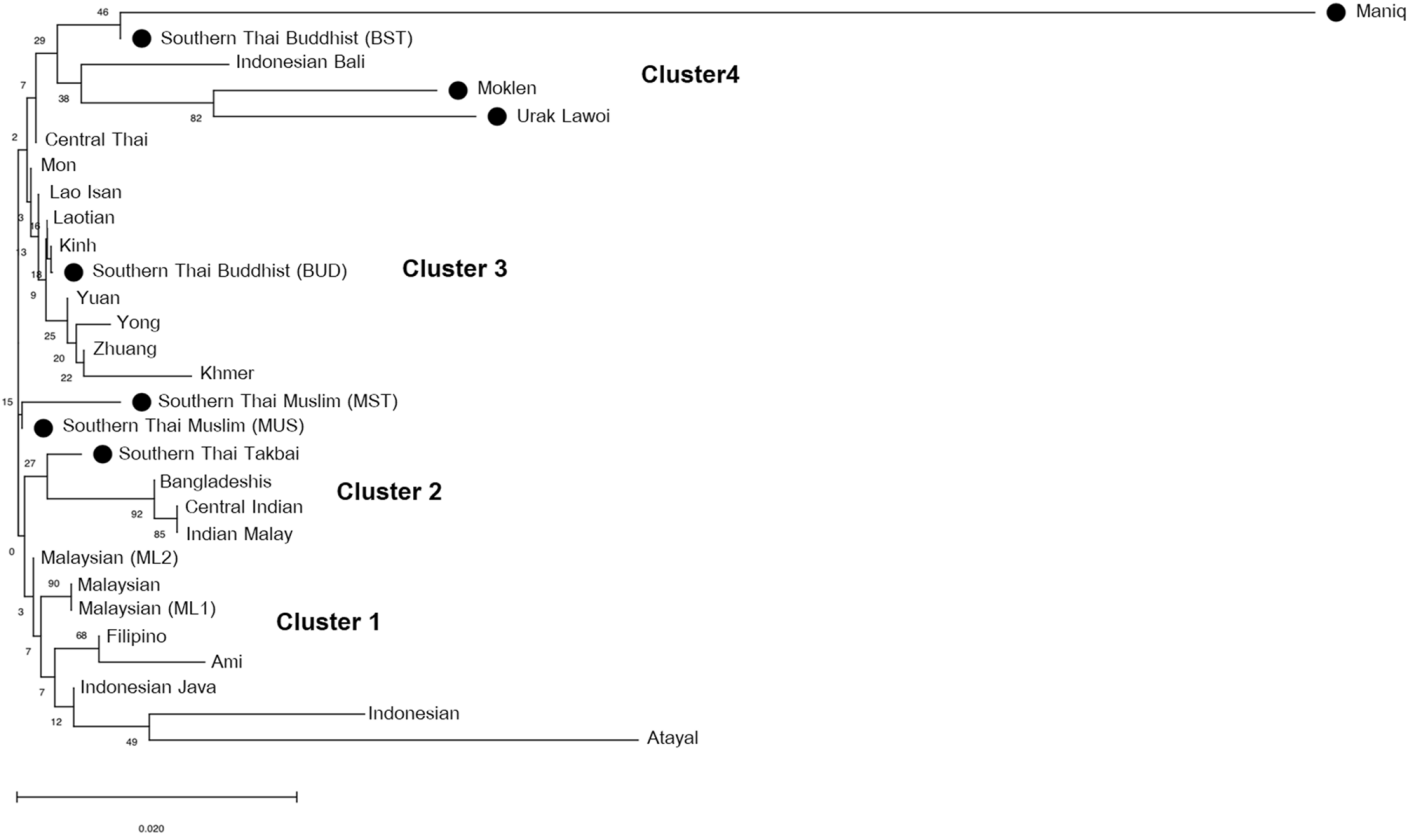
Neighbor-joining (NJ) tree. The NJ tree based on *F*_*st*_ computation from allele frequency of 15 STR loci from 29 populations, including southern Thai populations (indicated by dots) and other comparative Thai and Asian populations.

A neighbor-joining (NJ) tree based on allele frequencies of 15 STR loci among 29 Asian populations reveals four clusters of populations. Cluster 1 consists of populations from Island Southeast Asia and Malaysia while the South Asian populations occupy cluster 2. Cluster 3 comprises of Mainland Southeast Asian populations and cluster 4 belongs to the Thai sea nomads, Maniq from Thailand and Indonesians from Bali, with the extreme divergence of Maniq (Fig. 4). Interestingly, both southern Thai Muslim populations (MST and MUD) and southern Thai Takbai are positioned close to cluster 2 of South Asian. One southern Thai Buddhist population (BUD) is grouped with other Mainland Southeast Asian populations of cluster 3, while another southern Thai Buddhist population (BST) is clustered with southern Thai sea nomads in cluster 4 (Fig. 4). Several archaeological evidence indicated prehistorical contacts between India and present-day Thailand (and Cambodia) during the Iron Age that brought exotic goods and Buddhist and Hindu religions; early states in this area, e.g. Dvaravati in central Thailand and Langkasuka in Malay Peninsular were influenced by Indian cultures during initial establishment^5^. South Asian connections of southern Thai populations could be possibly driven by previous admixture, in agreement with previous study on genome-wide data^19^.

## Conclusion

We generated and analysed forensic STR loci in diverse ethnolinguistic groups from southern Thailand. In general, the Maniq and sea nomads are highly diverged from the other Thai groups, while the southern Thai populations are closer to the Malays and populations from central Thailand, reflecting different genetic structures of major Thais in each region that emphasize the importance of generating a database of allelic frequencies in southern regions of Thailand. Therefore, the allelic frequency generated here from combined STRs data from several populations is useful for further forensic investigation in the region. In anthropological genetic perspective, although the resolution of STRs to elucidate population history is lower than those of genome-wide data, several results here are concordant to previous genome-wide data, e.g. close relationship between southern and central Thais, reflecting certain usefulness of this set of markers. In addition, the Moklen and Urak Lawoi’ sea nomads have not been genetically investigated yet; this study initially provides basic genetic background of these enigmatic groups from southern Thailand. We found genetic distinction among Urak Lawoi’ and Moklen; the former had unique genetic perspective while the latter exhibited mixed ancestries, reflecting more population interaction with other populations. The limitations in this study is the limited sample size of the Moken which cannot be able to compare the results with other populations. Additional studies of sea nomads from other locations of southern Thailand coupled with further details from other genetic markers will be provided more insights into the genetic ancestry of AN speaking people in the Malay Peninsula.

## Materials and methods

### Sample

We newly collected 184 samples belonging to seven populations: AA-speaking Maniq, AN-speaking Moklen, Moken, Urak Lawoi’ and southern Thai Muslim and TK-speaking southern Thai Buddhist and southern Thai Takbai, using buccal swabs with written informed consent. Prior to the collection of samples, all volunteers were interviewed to screen for subjects unrelated for at least two generations. The rights of participants and their identity have been protected during the whole process of this research. All experiments were performed in accordance with relevant guidelines and regulations based on the experimental protocol on human subjects which was approved by the Khon Kaen University Ethic Committee (Protocol No. HE622223) and Naresuan University Institution Review Board (COA No. 0464/2017). When combined with previously published southern Thai Buddhist and southern Thai Muslim data^13^, this provides a total raw genotype data of 334 southern Thai samples (Table S1).

### Data collection

Genomic DNA was extracted from buccal swabs using the Gentra Puregene Buccal Cell Kit (Qiagen, Hilden, Germany) according to the manufacturer’s instructions. Each DNA sample was amplified for 15 STR loci in a multiplex PCR using a commercial AmpFlSTR Identifiler kit (Applied Biosystem, Foster City, CA, USA) according to the manufacturer’s protocols. The amplicons were genotyped by multi-capillary electrophoresis on an ABI 3130 DNA sequencer (Applied Biosystem), and allele calling was performed by the software GeneMapper v.3.2.1 (Applied Biosystem).

### Statistical analysis

**Figure 5:**
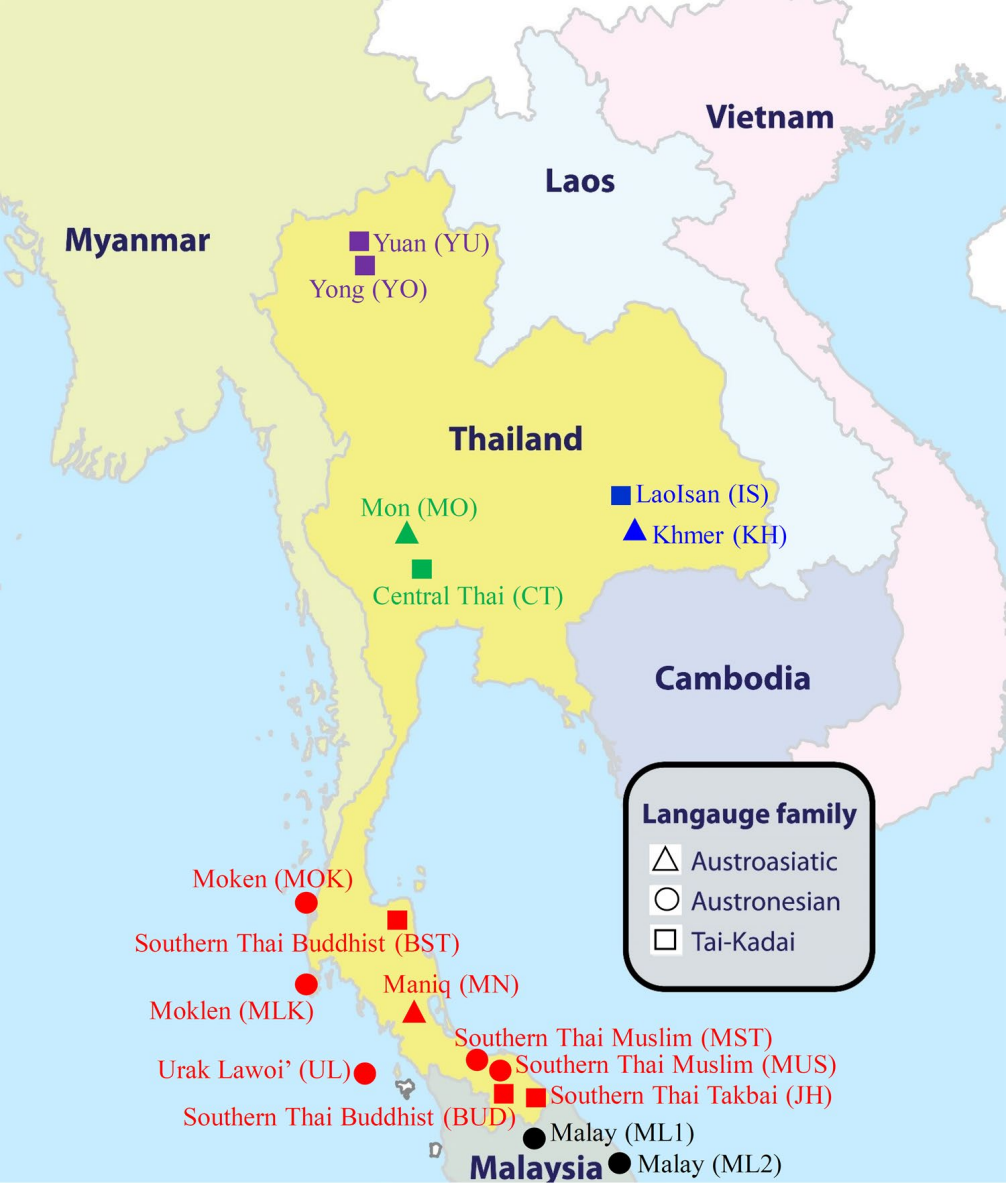
Map of the sampling locations of the 17 populations in analyses of genetic diversity and genetic structure, color-coded according to geographic region/country: red, purple, blue, green, and black indicating populations from southern Thailand, northern Thailand, northeastern Thailand, central Thailand and Malaysia, respectively while symbol-coded according to language family: Circle, square and triangle representing Austronesian, Tai-Kadai, Austroasiatic families, respectively. (Adob e Illustrat or CS4 14.0.0. http://www.adobe.com/sea/).

Arlequin v.3.5.2.2^21^ was used to calculate allele frequency, Hardy–Weinberg equilibrium (HWE) *P* values, observed heterozygosity (*H*_*O*_), expected heterozygosity (*H*_*E*_), total alleles, and gene diversity (GD). Significant levels for the HWE were adjusted according to the sequential Bonferroni correction (α = 0.05/15)^22^. We used the Excel PowerStats spreadsheet^23^ to compute several forensic parameters, including power of discrimination (PD), matching probability (MP), polymorphic information content (PIC), power of exclusion (PE), and typical paternity index (TPI) as well as the combined PD (CPD), combined MP (CMP), and combined PE (CPE). To reveal population relationships and population structures, we also combined genotyping data of additional eight populations from northern Thailand (Yuan and Yong), northeastern Thailand (Khmer and Lao Isan) and central Thailand (Mon and central Thai)^8,10–12,37^, and Malaysia (two Malay populations)^14^ (Table 1; Fig. 5). A genetic distance matrix based on sum of square difference (*R*_*st*_) was generated by Arlequin, and the matrix was then plotted in two dimensions by means of multidimensional scaling (MDS) using Statistica v.10 demo (StatSoft, Inc., USA). The heatmap visualization of *R*_*st*_ and MDS values were obtained using R package (R Development Core Team).

To delineate cryptic population structure using the Bayesian clustering method, we performed STRUCTURE version 2.3.4 under the following prior parameters: admixture, correlated allele frequencies, and assistance of sampling locations (LOCPRIOR model)^24–26^. We ran ten replications for each number of clusters (*K*) from 1 to 11 and used a burn-in length of 100,000 iterations, followed by 200,000 iteration running length. We used STRUCTURE Harvester^27^ to compute a second-order rate of change logarithmic probability between subsequent *K* values (*△K*) in order to identify the optimal *K* value in the data^16^. We used CLUMPAK^28^ and DISTRUCT^29^ to generate the final results of STRUCTURE. To evaluate genetic relatedness with other Asian populations, we used POPTREE v.2^30^ to generate a neighbor-joining (NJ) tree based on *F*_*st*_ computation by allele frequency of 15 STR loci of 29 populations from South and Southeast Asia^8,9,11,12,14,31–39^.

### Ethics statement

The rights of participants and their identity have been protected during the whole process of this research. All experiments were performed in accordance with relevant guidelines and regulations based on the experimental protocol on human subjects which was approved by the Khon Kaen University Ethic Committee (Protocol No. HE622223) and Naresuan University Institution Review Board (COA No. 0464/2017).

## Supplementary Information

Supplementary Table S1.

## Data Availability

Raw genotype data of 334 southern Thai samples are provided in Table S1.
